# Prognostic Power of Ensemble Learning in Colorectal Cancer with Peritoneal Metastasis: A Multi-Institutional Analysis

**DOI:** 10.3390/bioengineering13040434

**Published:** 2026-04-08

**Authors:** Yoshiko Bamba, Michio Itabashi, Hirotoshi Kobayashi, Kenjiro Kotake, Masayasu Kawasaki, Yukihide Kanemitsu, Yusuke Kinugasa, Hideki Ueno, Kotaro Maeda, Takeshi Suto, Kimihiko Funahashi, Heita Ozawa, Fumikazu Koyama, Shingo Noura, Hideyuki Ishida, Masayuki Ohue, Tomomichi Kiyomatsu, Soichiro Ishihara, Keiji Koda, Hideo Baba, Kenji Kawada, Yojiro Hashiguchi, Takanori Goi, Yuji Toiyama, Naohiro Tomita, Eiji Sunami, Yoshito Akagi, Jun Watanabe, Kenichi Hakamada, Goro Nakayama, Kenichi Sugihara, Yoichi Ajioka

**Affiliations:** 1 Department of Surgery, Institute of Gastroenterology, Tokyo Women’s Medical University, Tokyo 162-8666, Japan; 2 Department of Surgery, Teikyo University Hospital, Kanagawa 213-8507, Japan; 3 Department of Surgery, Sano City Hospital, Tochigi 327-0317, Japan; 4 Department of Surgery, Bell Land General Hospital, Osaka 599-8247, Japan; 5 Department of Colorectal Surgery, National Cancer Center Hospital, Tokyo 104-0045, Japan; 6 Department of Gastrointestinal Surgery, Institute of Science Tokyo, Tokyo 152-8550, Japan; 7 Department of Surgery, National Defense Medical College, Saitama 359-8513, Japan; 8 International Medical Center, Fujita Health University Hospital, Nagoya 470-1192, Japan; 9 Department of Gastroenterological Surgery, Yamagata Prefectural Central Hospital, Yamagata 990-2292, Japan; 10 Department of General and Gastroenterological Surgery, Toho University Omori Medical Center, Tokyo 143-8541, Japan; 11 Department of Colorectal Surgery, Utsunomiya Memorial Hospital, Tochigi 320-0013, Japan; 12 Department of Surgery, Nara Medical University, Nara 634-8521, Japan; 13 Department of Surgery, Osaka Rosai Hospital, Osaka 591-8025, Japan; 14 Department of Digestive Tract and General Surgery, Saitama Medical Center, Saitama Medical University, Saitama 350-0495, Japan; 15 Department of Gastroenterological Surgery, Osaka International Cancer Institute, Osaka 540-0008, Japan; 16 Department of Colorectal Surgery, National Center for Global Health and Medicine, Tokyo 162-8655, Japan; 17 Department of Surgical Oncology, The University of Tokyo Hospital, Tokyo 113-8655, Japan; 18 Department of Surgery, Teikyo University Chiba Medical Center, Chiba 299-0111, Japan; 19 Department of Gastroenterological Surgery, Graduate School of Medical Sciences, Kumamoto University, Kumamoto 860-8555, Japan; 20 Department of Gastrointestinal Surgery, Graduate School of Medicine, Kyoto University, Kyoto 606-8501, Japan; 21 Department of Surgery, Teikyo University School of Medicine, Tokyo 173-8605, Japan; 22 First Department of Surgery, University of Fukui, Fukui 910-8507, Japan; 23 Division of Reparative Medicine, Department of Gastrointestinal and Pediatric Surgery, Institute of Life Sciences, Mie University Graduate School of Medicine, Mie 514-8507, Japan; 24 Division of Cancer Treatment, Toyonaka Municipal Hospital, Osaka 560-8565, Japan; 25 Department of Surgery, Kyorin University School of Medicine, Tokyo 181-8611, Japan; 26 Department of Surgery, Kurume University School of Medicine, Kurume 830-0011, Japan; 27 Department of Surgery, Gastroenterological Center, Yokohama City University Medical Center, Kanagawa 232-0024, Japan; 28 Department of Gastroenterological Surgery, Hirosaki University Graduate School of Medicine, Aomori 036-8562, Japan; 29 Department of Gastroenterological Surgery (Surgery II), Nagoya University Graduate School of Medicine, Nagoya 466-8550, Japan; 30 Division of Molecular and Diagnostic Pathology, Graduate School of Medical and Dental Sciences, Niigata University, Niigata 950-2181, Japan

**Keywords:** colorectal cancer, peritoneal metastasis, cytoreductive surgery, hyperthermic intraperitoneal chemotherapy, machine learning, XGBoost, LightGBM, SHAP, LIME, prognosis

## Abstract

**Background:** Owing to significant clinical heterogeneity, the achievement of accurate survival forecasting for individuals with colorectal cancer and peritoneal metastasis continues to be a complex undertaking. We aimed to transcend traditional prognostic limitations by evaluating machine learning boosting models against standard regression-based methods in terms of estimating overall survival (OS). **Methods:** Utilizing a multi-institutional registry of 150 patients diagnosed with synchronous peritoneal metastasis of colorectal cancer, we integrated 124 clinicopathological variables to refine our predictive models. Beyond standard preprocessing—including standardization and median imputation—we rigorously compared XGBoost and LightGBM against Ridge, Lasso, and linear regression via five-fold cross-validation. To specifically address right-censoring, an XGBoost Cox model was implemented and validated using Harrell’s C-index, with SHAP and LIME providing essential model interpretability. **Results:** Boosting models consistently outperformed linear alternatives, which struggled with high error rates and negative R2 values. Specifically, XGBoost achieved an MAE of 475 ± 60 and an RMSE of 585 ± 88. The XGBoost Cox model reached a C-index of 0.64 ± 0.06. SHAP analysis highlighted inflammatory markers and peritoneal disease extent as the most influential prognostic drivers. **Conclusions:** While boosting models offer a clear accuracy advantage over linear methods, their prognostic power remains moderate. These findings underscore the potential of ensemble learning in oncology, yet mandate external validation before these tools can be integrated into clinical decision-making.

## 1. Introduction

Colorectal cancer remains one of the most frequently diagnosed malignancies worldwide and continues to be a major contributor to cancer-related mortality. Among its metastatic patterns, peritoneal dissemination represents a particularly aggressive phenotype associated with poor survival, even in the era of modern systemic therapy and advanced surgical strategies such as cytoreductive surgery and hyperthermic intraperitoneal chemotherapy [1,2,3,4,5]. An accurate estimation of overall survival is clinically important for treatment selection, perioperative decision-making, and patient counseling. Although conventional frameworks—such as TNM staging and clinicopathological scoring systems—provide structured prognostic information [1,4], they are primarily designed for population-level stratification and may not fully capture individual heterogeneity [6,7,8]. A novel Japanese classification system for colorectal peritoneal metastasis has recently been proposed to refine risk assessment [9].

With the growing availability of structured clinical data, machine learning techniques are increasingly being explored for outcome prediction in oncology [10,11,12,13,14,15,16]. Tree-based ensemble approaches, including gradient boosting algorithms such as XGBoost and LightGBM, are capable of modeling complex, non-linear relationships among variables [17,18]. However, concerns regarding interpretability remain a key obstacle to their integration into routine clinical practice. In contrast, regression-based models offer greater transparency but may be limited in handling high-dimensional and heterogeneous datasets.

While an increasing number of studies are utilizing artificial intelligence-based prognostic modeling for colorectal cancer, only some have systematically compared high-capacity ensemble methods with more interpretable regression approaches specifically in the context of peritoneal metastasis [19,20]. Therefore, the present study aimed to evaluate the prognostic power of machine learning-based survival modeling and to examine the balance between predictive performance and interpretability in this clinically complex population. Ultimately, this study seeks to provide a more precise tool for individualized risk stratification which could potentially assist in optimizing treatment selection and enhancing perioperative decision-making for patients with peritoneal metastasis.

## 2. Materials and Methods

### 2.1. Study Design

This investigation was conducted as a multi-center observational registry study under the auspices of the Japanese Society for Cancer of the Colon and Rectum (JSCCR). Twenty-eight affiliated institutions contributed data.

Patients who underwent surgical treatment for colorectal cancer with synchronous peritoneal metastases between October 2012 and December 2016 were eligible. A total of 150 patients were included. Clinical and pathological variables were recorded within three months after surgery, and survival outcomes were assessed three years postoperatively.

Formal written informed consent was secured from every patient enrolled in the study. In cases where peritoneal metastasis was identified intraoperatively, consent was obtained after surgery in accordance with institutional ethical policies. The research protocol received ethical authorization from both the JSCCR and the institutional review boards of all participating facilities.

Tumor location was categorized anatomically as follows: right-sided colon (appendix, cecum, ascending, transverse), left-sided colon (descending, sigmoid), and rectum (rectosigmoid [RS], upper rectum [Ra], lower rectum [Rb]).

### 2.2. Surgical Management

Surgical decision-making was not standardized within a predefined protocol due to the heterogeneity of disease presentation. The extent of primary tumor resection and management of peritoneal lesions were determined at the discretion of the attending surgeons based on intraoperative findings and overall clinical judgment.

### 2.3. Variables

A total of 124 demographic, laboratory, operative, and pathological variables were evaluated as potential predictors (Table 1).

### 2.4. Model Training and Evaluation

To examine prognostic modeling strategies, we evaluated tree-based ensemble algorithms (XGBoost and LightGBM) alongside regression-based approaches (Ridge, Lasso, and ordinary least squares regression).

#### 2.4.1. Data Processing

Continuous features were standardized prior to modeling. Qualitative variables underwent one-hot encoding prior to model training to facilitate their inclusion in the predictive analysis. Numerical gaps were filled using median imputation, while categorical variables were assigned a specific ‘Unknown’ status to account for missing information in a clinically plausible manner. All preprocessing procedures were performed prior to model fitting. Because imputation was not embedded within a fully nested cross-validation framework, a degree of inflation of the performance metrics cannot be excluded.

#### 2.4.2. Model Training

Hyperparameter optimization using Optuna was performed once using the full dataset prior to cross-validation. Subsequently, model development and performance estimation were conducted using five-fold cross-validation. Final model performance is presented as the mean ± standard deviation across folds. We acknowledge that this approach does not constitute a fully nested cross-validation framework and may introduce a degree of inflated performance metrics in performance estimates.

#### 2.4.3. Performance Metrics

Predictive accuracy for regression-based survival time prediction was assessed using mean absolute error (MAE), root mean square error (RMSE), and the coefficient of determination (R^2^). Visual evaluation included predicted-versus-observed plots, residual analysis, and error distribution histograms.

To appropriately account for right-censoring, we additionally implemented an XGBoost model using a Cox proportional hazards objective (“survival:cox”). Discriminative ability for time-to-event prediction was quantified using Harrell’s concordance index (C-index), and also estimated via five-fold cross-validation.

#### 2.4.4. Model Interpretability

Global and local interpretability were explored using SHAP (SHapley Additive exPlanations) for ensemble models and LIME for case-level explanations. For regression-based models, coefficient estimates were examined to assess feature influence.

## 3. Results

### 3.1. Patient Characteristics

A total of 150 patients were included in the analysis. Baseline characteristics are summarized in Table 2. The median age was 66 years [range, 30–89], and 56.0% of patients were male. The median BMI was 20.8 [range, 9.4–40.5] kg/m^2^. The most frequently observed comorbidities were hypertension (26.0%) and diabetes mellitus (14.0%). The primary tumor was located in the right colon in 53%, in the left colon in 29% and in the rectum in 18% of subjects. Median laboratory values included WBC 6800/μL, hemoglobin 11.6 g/dL, platelet count 30.5 × 10^4^/μL, albumin 3.6 g/dL, CRP 0.7 mg/dL, CEA 23.3 ng/mL, and CA19-9 48.5 U/mL. The median operative time was 191.5 min, and median blood loss was 123.5 mL. The median overall survival was 635 days [range, 21–2358]. Of the patients with synchronous peritoneal metastases, 30 (20%), 57 (38%), and 63 (42%) were classified as P1, P2, and P3, respectively, in accordance with the Japanese classification. The median PCI was 4 (range, 1–29). The median follow-up days of the surviving patients and median follow-up days of the deceased patients were 1364 days [range: 21–2340] and 535 days [range: 33–2358].

### 3.2. Predictive Performance

**Figure 1:**
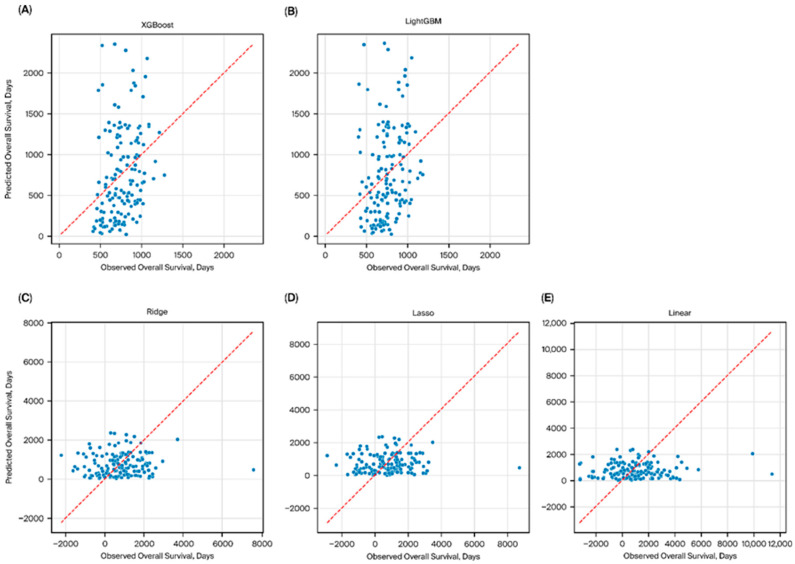
Predicted versus observed overall survival across five models. Scatter plots demonstrate the relationship between predicted and observed OS for XGBoost (**A**). LightGBM (**B**). Ridge regression (**C**). Lasso regression (**D**). Linear regression (**E**). The diagonal line indicates perfect prediction (y = x). Boosting models (**A**,**B**) showed closer alignment with the reference line, whereas linear models (**C**–**E**) exhibited large deviations and wider scatter, reflecting inferior predictive performance.

**Figure 2:**
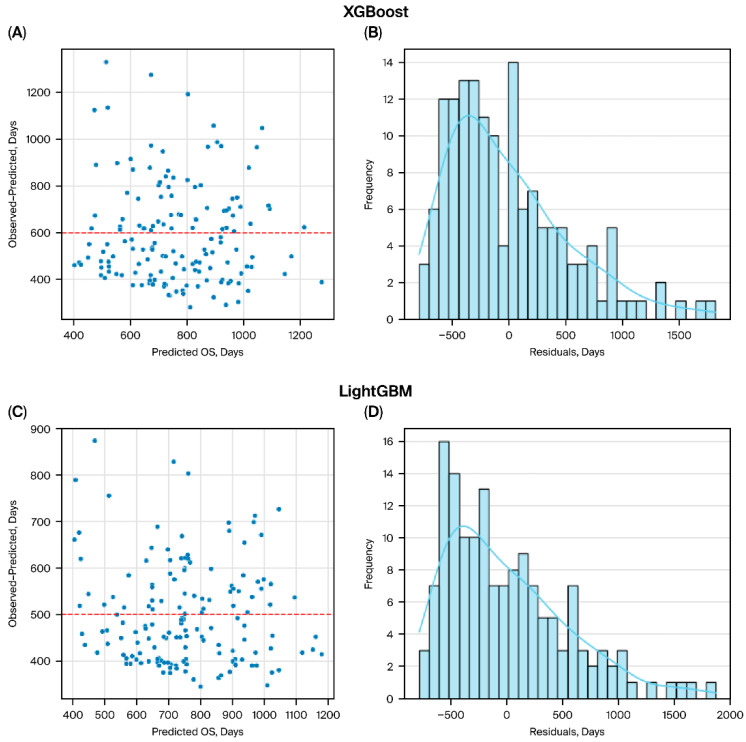

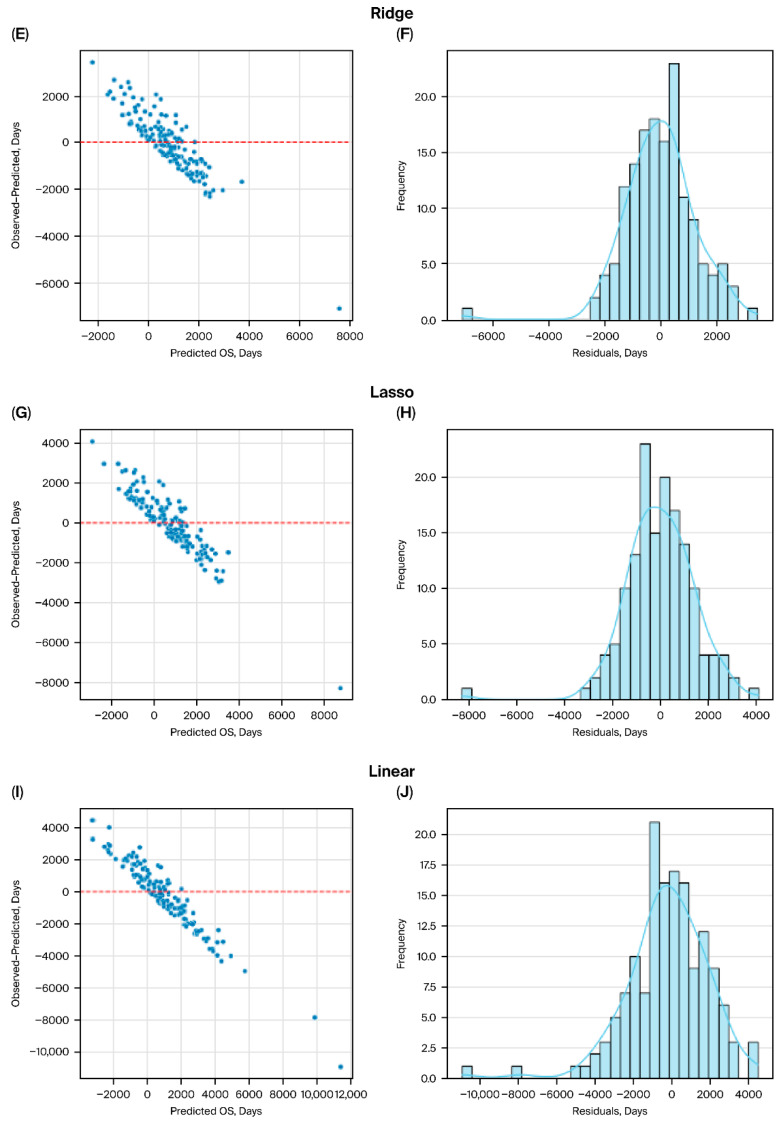
Residual and error distribution plots. Residual plots (left panels) and error distribution histograms (right panels) are shown for XGBoost (**A**,**B**), LightGBM (**C**,**D**). Ridge regression (**E**,**F**). Lasso regression (**G**,**H**). Linear regression (**I**,**J**). Boosting models (**A**–**D**) demonstrated residuals centered closer to zero and narrower error distributions, indicating better calibration and reduced bias. In contrast, linear models (**E**–**J**) exhibited wider and asymmetric error distributions, reflecting poorer predictive accuracy and systematic deviation from observed survival.

In this study, we compared high-performance machine learning models (XGBoost, LightGBM) with interpretable linear models (Ridge, Lasso, Linear regression) for predicting overall survival (Figure 1). Boosting models showed better regression performance than linear models. In five-fold cross-validation, regression-based survival time prediction showed limited performance (MAE 475 ± 60, RMSE 585 ± 88), with negative mean R^2^ values (−0.25 ± 0.30), indicating limited explanatory ability for precise survival time prediction. LightGBM showed comparable regression performance (Table 3). In contrast, linear approaches performed poorly, with high errors (MAE > 800, RMSE > 1100) and strongly negative R^2^ values, indicating a lack of predictive capability and potential overfitting (Table 3, Figure 2). In survival analysis using the Cox objective, the five-fold cross-validated C-index of the XGBoost model was 0.64 ± 0.06. For comparison with a conventional clinical index, a PCI-only Cox model achieved a five-fold cross-validated C-index of 0.62 ± 0.09. The XGBoost Cox model demonstrated slightly higher discrimination (0.64 ± 0.06), suggesting a modest incremental benefit from integrating multidimensional clinical variables beyond PCI alone.

These findings highlight that boosting methods, particularly XGBoost, can capture complex, non-linear patterns in survival data, whereas linear models fail to adequately reflect the prognostic heterogeneity of colorectal cancer with peritoneal metastasis. Although the absolute predictive accuracy remains modest, the results suggest that ensemble learning offers a more promising framework for survival prediction compared to traditional regression-based approaches.

### 3.3. Interpretability

**Figure 3:**
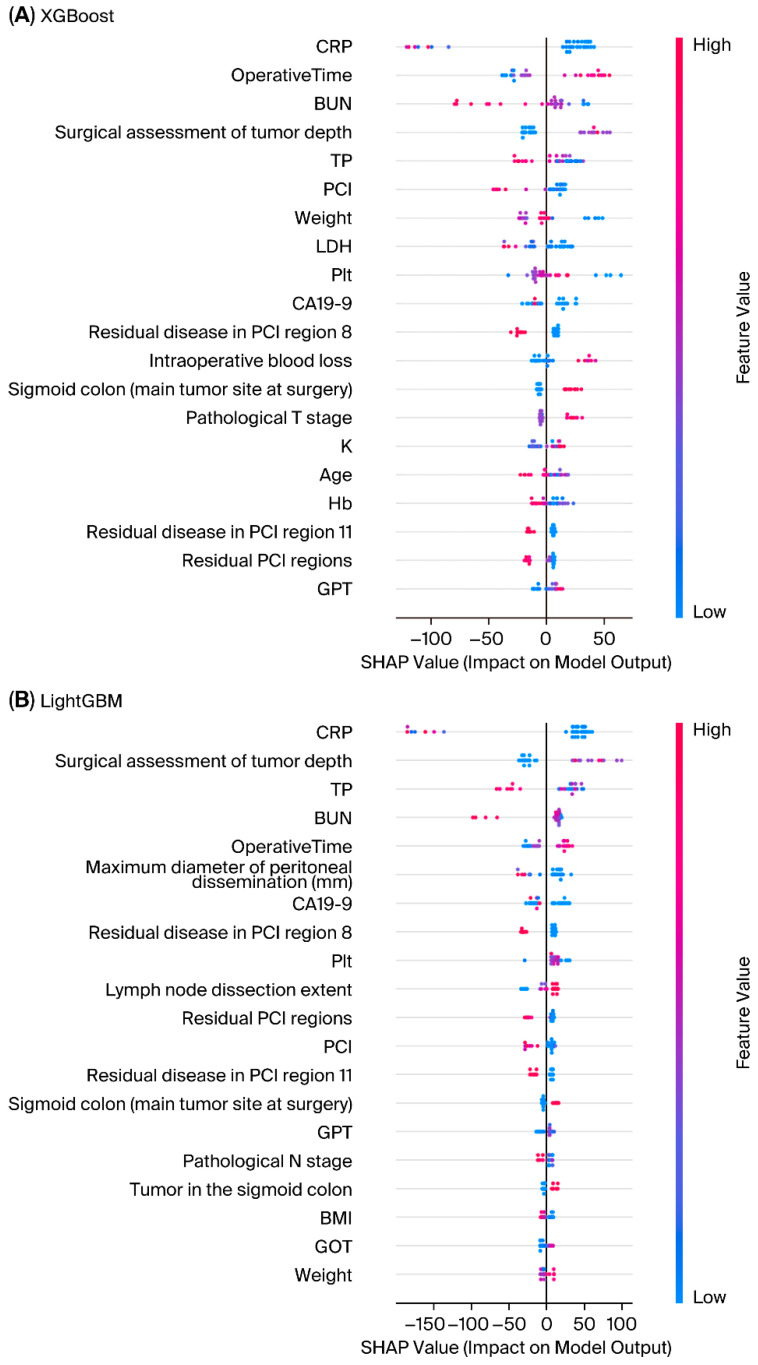
SHAP summary plots for boosting models. SHAP analysis identified the most influential predictors of overall survival. (**A**) XGBoost and (**B**) LightGBM. Each point represents an individual patient; the color denotes the feature value (red = high, blue = low), and the horizontal position reflects the magnitude and direction of its contribution to the prediction. Both models consistently highlighted clinically established prognostic factors, supporting interpretability and alignment with clinical knowledge.

Global feature importance derived from SHAP analysis is shown in Figure 3. Both XGBoost and LightGBM identified systemic inflammation markers (CRP, BUN), surgical assessment of tumor depth, and peritoneal metastasis-related variables (e.g., residual PCI regions, dissemination diameter) as key determinants of overall survival, with operative time and CA19-9 further contributing to prediction.

**Figure 4:**
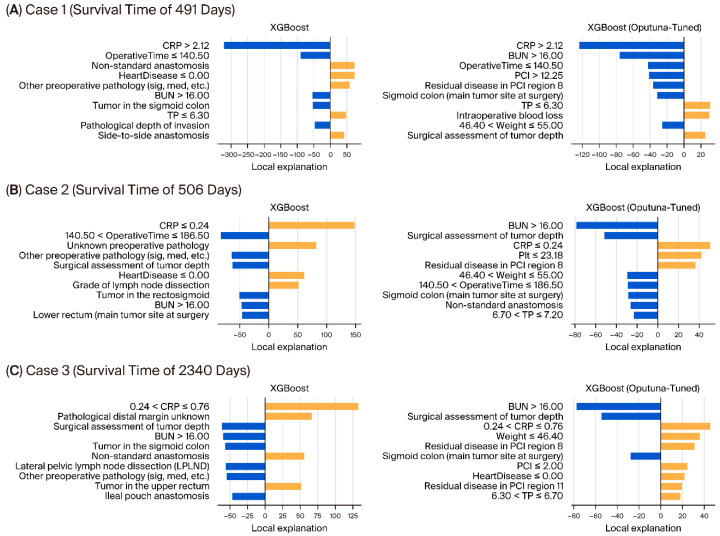
LIME explanations for individual survival predictions using XGBoost models. Local explanations of three representative cases (**A**–**C**) are shown for both standard XGBoost and Optuna-tuned XGBoost models. Each bar indicates the contribution of a clinical or pathological feature to the survival prediction: green bars represent positive contributions (prolonging survival), while red bars represent negative contributions (shortening survival). Case A (491 days), Case B (506 days), and Case C (2340 days) illustrate differences in the relative importance of preoperative, intraoperative, and pathological variables between models.

Case 1 (survival time: 491 days) (Figure 4): Increased CRP levels, elevated BUN, and extended operative duration emerged as significant determinants of poor prognosis in both models. Conversely, sigmoid colon primary tumors and lower TP concentrations were associated with favorable survival. Specifically, the Optuna-optimized XGBoost model pinpointed residual disease (PCI region 8) and intraoperative hemorrhage as additional clinical indicators of diminished survival.

Case 2 (survival time: 506 days) (Figure 4): In this instance of intermediate survival, the model’s prediction was bolstered by favorable markers such as low CRP and the absence of cardiac comorbidities. These were countered by the negative impacts of elevated BUN, deep surgical invasion, and residual dissemination. Analysis via the Optuna-tuned model further underscored body weight, operative duration, and overall PCI burden as pivotal prognostic drivers.

Case 3 (survival time: 2340 days) (Figure 4): This long-term survivor was characterized by a constellation of positive factors, including baseline low CRP, complete resection of dissemination, and favorable perioperative parameters. Despite adverse features such as advanced tumor depth and high BUN, the model correctly identified the lack of residual disease in PCI regions as a dominant survival determinant. These case-specific insights illustrate how local interpretability tools can bridge the gap between algorithmic outputs and clinical reality, offering a transparent weighing of competing prognostic features to validate the model’s plausibility.

## 4. Discussion

This multi-institutional study involved the development and comparative assessment of machine learning-based survival models tailored for patients diagnosed with colorectal peritoneal metastasis. Our findings highlight that boosting-based ensemble models, particularly XGBoost [14], demonstrated relatively better predictive performance compared to traditional regression models, although the absolute predictive power remained modest. Linear approaches, including Ridge, Lasso, and standard regression [12,13], showed higher prediction errors and negative R^2^ values, highlighting the limitations of linear modeling in this heterogeneous population.

The improved performance of boosting models likely reflects their proficiency in modeling multifaceted, non-linear interactions between clinical, pathological, and perioperative variables and survival outcomes. SHAP analysis [12] further confirmed that key prognostic factors identified by the models—such as peritoneal dissemination grade [7,8], CA19-9, operative time, and nodal status—are consistent with established clinical knowledge. Recent studies further demonstrated that SHAP enables both global and local interpretations of tree-based ensemble models, facilitating cautious integration into clinical research contexts [15]. LIME analyses [13] provided case-level interpretability, demonstrating how individual predictions were influenced by specific patient factors. These synergistic methods integrate machine learning outputs with clinical interpretability, which is a vital prerequisite for translational research in oncology [10,11]. However, the marginal improvement of boosting models over linear methods may primarily reflect their inherent algorithmic flexibility rather than a definitive capture of the underlying biological complexity. The overall low predictive power suggests that standard clinicopathological variables available in current registries may lack the necessary molecular or biological depth to fully explain survival dynamics in this highly heterogeneous population. This indicates that the prognostic signal within conventional clinical data may be reaching its ceiling, and further gains in accuracy will likely require the integration of genomic or molecular biomarkers [21].

Regression-based survival time prediction showed limited explanatory ability under cross-validation, reflecting the intrinsic difficulty of precise time-to-event prediction in this heterogeneous population. In contrast, rank-based survival modeling demonstrated moderate discriminative ability (C-index 0.64), suggesting that relative risk stratification may represent a more appropriate framework in this setting. Existing clinical prognostic systems for colorectal peritoneal metastasis primarily rely on PCI and the completeness of cytoreduction. In our cohort, PCI alone demonstrated moderate discrimination (C-index 0.62). The XGBoost Cox model achieved a slightly higher C-index (0.64), indicating modest incremental improvement in discrimination, although the absolute gain was limited.

In line with these observations, an increasing body of literature is demonstrating the advantage of boosting-based ensemble models for survival prediction in oncology. Comparative studies using large registry-based datasets have shown that gradient boosting approaches, including XGBoost [14] and LightGBM [22], consistently outperform conventional Cox proportional hazards models and linear regression-based methods in terms of discrimination and overall predictive performance [23,24]. These findings suggest that the ability of boosting algorithms to model non-linear effects and complex interactions among heterogeneous clinical variables is particularly advantageous in advanced cancer populations, where prognosis is driven by multifactorial and interdependent processes [25,26,27]. Importantly, although the performance gains reported in these studies are generally modest, their consistent reproducibility across different cancer types and datasets supports the robustness and generalizability of boosting-based survival models [25,26,27]. In this context, the extremely large errors and highly negative R2 values observed not only for Lasso but also for linear regression and, to a lesser extent, Ridge regression likely reflect instability of linear models in a high-dimensional setting with a limited sample size. Given the near one-to-one ratio between predictors and patients, these models may fail to estimate stable coefficients, particularly in the presence of multicollinearity and weak signal. This results in poor generalization performance and exaggerated error metrics.

Clinically, these findings suggest that boosting models could be applied as adjunctive tools for risk stratification, particularly when combined with interpretability methods that align predictions with known prognostic markers. For instance, SHAP analysis emphasized peritoneal dissemination grade and CA19-9—both widely accepted prognostic factors [7,8]—thereby increasing clinician confidence in model outputs [28]. Nevertheless, until predictive accuracy is further refined, these models should serve as a complement to, rather than a replacement for, conventional prognostic tools [6].

We acknowledge several limitations to our study. First, the high dimensionality of our data—with 124 predictors for a cohort of 150 patients—poses an inherent risk of overfitting. To mitigate this issue, we applied regularization within boosting algorithms and evaluated model performance using five-fold cross-validation, presenting mean ± standard deviation across folds to ensure transparency regarding stability. Despite these efforts, the small sample size remains a significant limitation. Second, hyperparameter optimization using Optuna was performed once using the full dataset prior to cross-validation, rather than within a fully nested cross-validation framework. This approach may introduce a degree of optimistic bias in performance estimation. Third, preprocessing steps, including missing value imputation, were conducted prior to cross-validation and were not embedded within a nested framework, which may further contribute to optimistic bias.

Fourth, the study period (2012–2016) represents a potential limitation. Treatment strategies for colorectal peritoneal metastasis have evolved over time, including through advances in systemic chemotherapy and perioperative management. Therefore, temporal changes in clinical practice may influence survival outcomes and limit the generalizability of the present findings to contemporary cohorts. Fifth, molecular and genomic variables were not included in the present analysis. The integration of multi-omics or biologically enriched datasets may improve predictive accuracy in future studies. Sixth, external validation using an independent cohort was not available. Therefore, the current findings should be considered exploratory and hypothesis-generating. Independent and temporally distinct validation cohorts are required before clinical application. Finally, the limited regression-based predictive performance observed under cross-validation indicates that larger and more comprehensive datasets will be necessary to achieve clinically actionable prognostic models. Furthermore, we acknowledge that the predictive superiority of ensemble learning observed in this study is relative. The modest absolute performance underscores a fundamental limitation of the predictor variables themselves; without high-resolution molecular or genomic data, even advanced machine learning algorithms struggle to account for the stochastic nature of peritoneal metastasis. Our results thus serve as a quantitative benchmark for the current limits of registry-based survival prediction, highlighting the necessity for future multi-omic data integration. Additionally, as this study includes data from 28 institutions, potential inter-institutional heterogeneity in treatment strategies, surgical indications, and patient selection may have influenced the results and should be considered a limitation.

Taken together, our findings indicate that ensemble learning methods such as XGBoost [14] and LightGBM [18] exhibit superior prognostic power compared to traditional linear regression models in this clinical setting. While predictive accuracy remains limited, the combination of boosting algorithms with interpretability tools such as SHAP [12] and LIME [13] provides a promising framework for future prognostic modeling. By leveraging the prognostic power of these high-capacity models, clinicians can gain more granular insights into survival dynamics. Further research with larger, genomically enriched datasets and external validation is warranted to refine these models and advance their clinical utility.

## 5. Conclusions

In summary, boosting-based ensemble techniques—specifically XGBoost and LightGBM—demonstrated superior prognostic power compared to traditional linear regression in predicting overall survival for patients suffering from peritoneal metastasis of colorectal origin. While absolute predictive power was moderate, the integration of SHAP and LIME ensured clinical relevance, providing a transparent rationale by aligning model outputs with established prognostic factors. Ultimately, our findings highlight ensemble-based algorithms as sophisticated adjuncts that can enhance the prognostic power of existing frameworks in clinical oncology. To advance these models toward clinical translation, future studies incorporating larger cohorts, multi-omics markers, and multi-center external validation are essential.

## Figures and Tables

**Table 1:** Variables iIncluded in the aAnalysis.

Category	Variables Included
Demographics and Clinical Background	Age; Sex; PS; Height; Weight; BMI; Surgery history; Comorbidities (DM, HT, heart disease, cerebrovascular disease)
Laboratory data	WBC; RBC; Hb; Ht; Plt; TP; Alb; T-bil; ALP; AST; ALT; LDH; BUN; Cr; Na; K; Cl; CRP; CEA; CA19-9
Tumor Location	Cecum (C); Appendix (A); Transverse (T); Descending (D); Sigmoid (S); Rectosigmoid (RS); Upper rectum (Ra); Lower rectum (Rb)
Preoperative Treatments	Preoperative blood transfusion (BTF); Supportive treatment
Preoperative Pathology	Tub1; Tub2; Por; Muc; Adenosquamous; Other; Unknown
Operative Variables	Main tumor site; Primary site removal; Laparoscope; HIPEC; Operative time; Blood loss; Intraoperative transfusion; Residual disease; Resected organs; Lymph node dissection; Lateral pelvic lymph node dissection; ascites; anastomosis methods; surgical tumor depth; PCI (Count/Diameter); residual PCI (Count/Diameter); Complications (ileus, dehiscence, embolism)
Postoperative Pathology	Pathological TNM; lymph invasion (ly); venous invasion (v); Curability; Pathological PM/DM/RM
Outcomes	Overall survival (days)

Abbreviations: tub1, well-differentiated tubular adenocarcinoma; tub2, moderately differentiated tubular adenocarcinoma; por, poorly differentiated adenocarcinoma; muc, mucinous adenocarcinoma. PM, proximal margin; DM, distal margin; RM, radial margin.

**Table 2:** Patient characteristics.

Variable	n (%) or Median [Range]
Age (years)	66 [30–89]
Gender	
Male	84 (56)
Female	66 (44)
BMI	20.8 [9.4–40.5]
Comorbidity	
Hypertension	39 (26.0)
Diabetes mellitus	21 (14.0)
Heart disease	16 (10.7)
Cerebrovascular disease	6 (4.0)
Location of primary tumor	
Right colon	79 (53)
Left colon	44 (29)
Rectum	27 (18)
Laboratory data	
WBC (/μL)	6800 [3000–13,500]
Hemoglobin (g/dL)	11.6 [5.9–16.9]
Platelet (×10^4^/μL)	30.5 [6.4–71.5]
Albumin (g/dL)	3.6 [1.8–4.7]
CRP (mg/dL)	0.7 [0.0–25.4]
CEA (ng/mL)	23.3 [0.7–15,000.0]
CA19-9 (U/mL)	48.5 [0.4–22,599.1]
Histologic type of primary tumor	
tub1 or tub2	110 (73)
Others	36 (24)
Unknown	4 (3)
Primary tumor resection	
Absent	24 (16)
Present	126 (84)
Operative time (min)	191.5 [30–719]
Bleeding (mL)	123.5 [0–2856]
Histologic type of primary tumor	
Well or mod	110 (73)
Others	36 (24)
Unknown	4 (3)
Residual tumor	
R0	32 (21)
R1	5 (3)
R2	113 (75)
T-category	
T3	12 (8)
T4a	86 (57)
T4b	28 (19)
Unknown	24 (16)
N-category	
N0	29 (19)
N1a	18 (12)
N1b	24 (16)
N2a	27 (18)
N2b	28 (19)
Unknown	24 (16)
Peritoneal metastasis	
P1	30 (20)
P2	57 (38)
P3	63 (42)
PCI	4 [1–29]
Outcome	
Death	121 (80.7)
Survival	29 (19.3)
Lifetime days (day; overall survival)	635 [21–2358]
Median follow-up days of the surviving patients	1364 [21–2340]
Median follow-up days of the dead patients	535 [33–2358]

Abbreviations: BMI, body mass index; WBC, white blood cell; CRP, C-reactive protein; CEA, carcinoembryonic antigen; OS, overall survival; PCI, peritoneal cancer index. Peritoneal metastasis: PX: Peritoneal metastasis cannot be assessed. P0: No peritoneal metastasis. P1: Metastasis localized to adjacent peritoneum. P2: Limited metastasis to distant peritoneum. P3: Diffuse metastasis to distant peritoneum.

**Table 3:** Model performance comparison (five-fold cross-validation, mean ± SD).

Model	MAE	RMSE	R^2^
XGBoost	475 ± 60	585 ± 88	−0.25 ± 0.30
LightGBM	504 ± 52	623 ± 63	−0.43 ± 0.32
Ridge Regression	893 ± 97	1145 ± 206	−3.77 ± 1.03
Lasso Regression	3458 ± 1694	4944 ± 3145	−99.95 ± 96.71
Linear Regression	3972 ± 1578	5468 ± 2621	−119.62 ± 80.42

MAE = Mean Absolute Error. RMSE = Root Mean Squared Error. R^2^ = Coefficient of Determination.

## Data Availability

Ethical and institutional constraints preclude the public deposition of our datasets. Nevertheless, researchers may access the data by submitting a reasonable request to the corresponding author, contingent upon formal authorization from the JSCCR.

## Abbreviations

BMI	body mass index
WBC	white blood cell
CRP	C-reactive protein
CEA	carcinoembryonic antigen
OS	overall survival
MAE	mean absolute error
RMSE	root mean square error
